# Redirecting emergency medical services patients with unmet primary care needs: the perspective of paramedics on feasibility and acceptance of an alternative care path in a qualitative investigation from Berlin, Germany

**DOI:** 10.1186/s12873-022-00660-2

**Published:** 2022-06-11

**Authors:** Sarah Oslislo, Lisa Kümpel, Rebecca Resendiz Cantu, Christoph Heintze, Martin Möckel, Felix Holzinger

**Affiliations:** 1 Charité - Universitätsmedizin Berlin, corporate member of Freie Universität Berlin and Humboldt-Universität zu Berlin, Institute of General Practice, Charitéplatz 1, 10117 Berlin, Germany; 2 Charité - Universitätsmedizin Berlin, corporate member of Freie Universität Berlin and Humboldt-Universität zu Berlin, Division of Emergency Medicine, Campus Mitte and Virchow, Charitéplatz 1, 10117 Berlin, Germany

**Keywords:** Acute care pathways, Emergency medical services, Emergency department, Primary care, Outpatient, Acceptability, Feasibility, Qualitative research, Interview

## Abstract

**Background:**

Against the backdrop of emergency department (ED) overcrowding, patients’ potential redirection to outpatient care structures is a subject of current political debate in Germany. It was suggested in this context that suitable lower-urgency cases could be transported directly to primary care practices by emergency medical services (EMS), thus bypassing the ED. However, practicality is discussed controversially. This qualitative study aimed to capture the perspective of EMS personnel on potential patient redirection concepts.

**Methods:**

We conducted qualitative, semi-structured phone interviews with 24 paramedics. Interviews were concluded after attainment of thematic saturation. Interviews were transcribed verbatim, and qualitative content analysis was performed.

**Results:**

Technical and organizational feasibility of patients’ redirection was predominantly seen as limited (theme: “feasible, but only under certain conditions”) or even impossible (theme: “actually not feasible”), based on a wide spectrum of potential barriers. Prominently voiced reasons were restrictions in personnel resources in both EMS and ambulatory care, as well as concerns for patient safety ascribed to a restricted diagnostic scope. Concerning logistics, alternative transport options were assessed as preferable. Regarding acceptance by stakeholders, the potential for releasing ED caseload was described as a factor potentially promoting adoption, while doubt was raised regarding acceptance by EMS personnel, as their workload was expected to conversely increase. Paramedics predominantly did not consider transporting lower-urgency cases as their responsibility, or even as necessary. Participants were markedly concerned of EMS being misused for taxi services in this context and worried about negative impact for critically ill patients, as to vehicles and personnel being potentially tied up in unnecessary transports. As to acceptance on the patients’ side, interview participants surmised a potential openness to redirection if this would be associated with benefits like shorter wait times and accompanied by proper explanation.

**Conclusions:**

Interviews with EMS staff highlighted considerable doubts about the general possibility of a direct redirection to primary care as to considerable logistic challenges in a situation of strained EMS resources, as well as patient safety concerns. Plans for redirection schemes should consider paramedics’ perspective and ensure a provision of EMS with the resources required to function in a changing care environment.

**Supplementary Information:**

The online version contains supplementary material available at 10.1186/s12873-022-00660-2.

## Background

Recent statistics from several international settings have shown increasing numbers of medically unnecessary EMS transports [1, 2]. In this context, redirection of non-urgent patients to other care structures to mitigate ED overcrowding is discussed [3, 4]. Studies from the United Kingdom [5] and Sweden [6] estimated potential redirection at 12 to 16% of EMS caseload. In Germany, it is claimed that up to one third of EMS cases are not emergency patients in the classical sense [7]. Calculations from the United States suggest high potential cost savings for patient redirection to more appropriate outpatient care settings, while concurrently improving care continuity [8]. However, systematic reviews on interventions to reduce ED utilization have shown mixed results and appraised current evidence for effectiveness as insufficient [9, 10].

Redirecting less urgent acute patients with suspected non-hospital care needs is also discussed in German national health politics currently. A 2018 expert opinion by the Advisory Council on the Assessment of Developments in the Health Care System [11] suggests a direct redirection of less urgent EMS cases to regular ambulatory care practices, e.g. primary care (PC) providers, thus bypassing the ED. However, this notion is quite controversial. Firstly, it would require the establishment of organizational cooperation pathways between EMS and physicians’ governing bodies (Associations of Statutory Health Insurance Physicians). Secondly, recent detailed analyses of case characteristics have cast doubt on the realistic scope for redirection. While a retrospective evaluation of prehospital and clinical data in Berlin [12] has indicated that one out of ten EMS patients in ED could be potentially redirected to non-hospital care, a 2021 study of respiratory ED patients [13] estimated the redirection potential at only 2 % of all EMS cases if considering not only medical data, but also patients’ personal judgment regarding the appropriate care setting.

Predominantly however, studies on redirection of EMS utilizers rely on secondary data and expert opinions and lack a broader perspective on these cases. Filtering by mere medical criteria neglects both organizational barriers and the question of acceptance by patients and involved health care professionals. Both aspects – feasibility and potential acceptance – are prerequisite to successful implementation of any new care pathway, and this remains a topic scarcely touched in the available literature on EMS redirection potential. In a conceivable redirection scheme for less urgent EMS patients, the actual decision to redirect patients to non-ED ambulatory care would lie with the responding EMS personnel. Thus, their views on feasibility and acceptance of redirection notions are paramount to understanding potential obstacles to an implementation of such new care pathways, and their daily experience with EMS utilizers in the “real life” pre-clinical care environment is potentially invaluable for determining actual practicability. However, quantitative investigations are ill suited to grasp the complexity of issues and considerations involved. Here we thus present the results of the qualitative part of the EMAPREPARE study (Emergency and Acute Medicine - Primary Care Demands in Patients resorting to Emergency Departments), which consisted of interviews with paramedics to gain deeper insights into their views and perceptions regarding the feasibility and acceptance of a redirection pathway corresponding to the process suggested by the German Government Advisory Council.

## Methods

### EMAPREPARE study

The qualitative interviews are a component of the mixed-methods study EMAPREPARE, part of the Berlin health care research network EMANet (Emergency and Acute Medicine Network for Health Care Research Berlin).

EMAPREPARE focuses on ED patients presumed potentially suitable for outpatient care, and thereby investigates whether and to which extent this applies to patients transported by EMS. This question is addressed by 1) the qualitative paramedic interviews reported here, and 2) a currently ongoing quantitative survey of EMS patients in three emergency departments in the central district of Berlin (Berlin-Mitte). Reporting for the qualitative data presented in this paper is based on the COREQ criteria [14]. The core research team for this investigation consisted of S.O. (health scientist experienced in qualitative research), L.K. (health scientist and PhD candidate), R.R.C. (ED physician), and F.H. and C.H. (general practitioners and senior researchers). Researchers had no organizational, personal, or financial connection to the Advisory Council. The EMAPREPARE study is registered in the German Clinical Trials Register (ID DRKS00023480, registration date 27/11/2020).

### Study setting

The study was conducted in the federal city state of Berlin, Germany. Berlin is Germany’s capital and largest city and represents a highly urbanized environment. All participants were personnel of the Berlin Fire Brigade, which is the responsible entity providing EMS services in the city state and has about 4500 employees [15]. For some Fire Brigade paramedics with a double qualification, professional duties also include firefighting and technical assistance duties. In EMS, there are different qualification levels, of which “Notfallsanitäter” (“emergency paramedics”) represent the most qualified non-physician staff with three years of training, while the “Rettungssanitäter” constitutes a lower-level certification attained after a few months of training. The Fire Brigade’s operational volume in 2020 amounted to more than 350.000 EMS alerts in the Berlin catchment area of 3.7 million inhabitants [15]. In the German health care system, patients can alert EMS via a hotline (112) by which a dispatch centre is reached. Dispatch then initiates deployment of a vehicle staffed by paramedics, with optional additional assignment of an emergency physician, depending on presumed urgency and severity. For organizational as well as forensic reasons, EMS alerts are comparably rarely passed on to on-call ambulatory care providers, and ambulance deployment is the rule. Hereafter, patients are almost invariably transported to a hospital ED by EMS, as exclusive on-site care without ensuing transport is not covered by current reimbursement schemes in the German Statutory Health Insurance system and there neither is a formalized redirection scheme to non-hospital service providers. Patients themselves are not charged for EMS services, as these are covered by insurance. Neither are there any penalty fees if patients alert EMS for non-urgent complaints. For such cases, the association of Statutory Health Insurance Physicians offers a house call service, but this is accessible through a hotline (116117) distinct from EMS dispatch. Thus, patients basically decide on their own which call centre they alert, depending on their subjective judgment of urgency. Recent health care reform plans include a fusion of dispatch structures of on-call physicians and EMS, but this has not been realized currently.

### Interview guide

Interviews were conducted using a semi-structured interview guide, thematically based on our research interests. Topics and questions were refined in discussion with emergency physicians and additionally agreed with stakeholders in the Berlin Fire Brigade. The guide featured a great degree of openness and centred on larger topics rather than containing a list of compartmentalized pre-formulated questions, leaving both interviewers and interviewees a lot of freedom and allowing flexible situational adaption to the conversation flow [16]. The first three interviews were part of a pilot test for the interview guide. Pilot interviews were discussed in the research team and the guide was finalized after revision of a question and adding of another one. Additional to the redirection topic covered in this article, the interview guide included some questions on potentially unmet primary care needs in EMS patients. Table 1 provides a snapshot of the questions pertinent to the topics addressed here. The entire interview guide is available in Additional file 1.

**Table 1:** Excerpt from the interview guide

• What do you think about the redirection model proposed by the Government Advisory Council?	
• How do you assess the technical and organizational feasibility of this pathway? Could you give reasons for this judgment?	
• How do you appraise the acceptability by EMS staff/patients? Could you give reasons for this judgment?	

### Recruitment and data collection

A convenience sampling strategy was used, with interviewees recruited via EMS administration (Fire Brigade), which circulated information about the study by e-mail to their staff, as well as direct address of paramedics in the participating EDs by the EMAPREPARE study nurses. This double-tracked recruitment approach was chosen due to EMS administration expecting limited preparedness to volunteer for such interviews as to the high workload of their personnel. For this reason, it was also chosen to refrain from applying theoretical sampling criteria [17]. However, only interview partners could be selected whose daily work did touch decision-making on patient transport and pre-clinical care. This did apply to paramedics in regular clinical EMS duty, as well as persons working in EMS dispatch, but not to exclusively managerial positions or staff engaged in firefighting only. Participants were informed about the study aims and the interviewers’ backgrounds, and written informed consent was obtained prior to the interviews. When it came apparent in the later course of the interviewing stage that only male interview partners had volunteered to participate so far, remaining recruitment efforts were focused on female EMS staff to reduce potential sample bias. We thus managed to add several female participants to the sample in the final stage, but the preponderance of male participants still mirrors the higher share of men employed in German EMS [18]. Recruitment efforts were concluded when collected material suggested content saturation by redundancies in topics and aspects expressed [19].

Interviews were conducted between July 2021 and February 2022 (S.O. & L.K.), at off-duty times. Interviewees did not receive any financial compensation for participating. Due to the COVID-19 pandemic, participants were interviewed by telephone to avoid potential infection risk. Interviews were audio-recorded. Characteristics of participants were documented after the interviews. The material was transcribed verbatim, and transcript data was pseudonymized. Repeat interviews were not carried out. Eventual special interview circumstances (disturbances etc.) were documented for context.

### Data analysis

Qualitative content analysis with a combined deductive-inductive approach was used [20]. Content analysis with a systematic coding procedure was deemed the most suitable evaluation method for answering the research question by focusing on essential topics. Researchers deductively developed an initial coding tree based on the interview guide. Additional codes and categories were then inductively derived directly from the interview transcripts and included into the code tree. The analysis was carried out simultaneously to the interview process. A third of the material was independently coded by two researchers (S.O. & L.K.) and codes were collated [21] by comparing and discussing similarities and discrepancies and adapting the coding tree accordingly. Differences were resolved by consensus or by involvement of a third research team member if agreement was not attainable. Double-coding did show increasing convergence of coding and was then concluded. Coding and interpretation were continually discussed in the core research team, which developed consensus definitions for all codes. The revised code tree was then applied to the whole material, including re-coding of data viewed earlier in the process. Codes were subsequently condensed to themes, which were initially proposed by the coder and discussed in the research team by jointly reflecting sample citations and discussing their representation by the potential category and possible alternatives. Discussions in the team also reflected potential influences of researchers’ beliefs and experiences on categorization and interpretation. Discussions and revisions of themes, coding etc. were documented. We did also present and discuss the results with representatives of EMS (both staff and management) as well as colleagues working in the ED, and their external views and feedback did aid interpretation. A member check was not performed. The software MAXQDA 2020 was used for transcribing, coding, and analysis.

## Results

Via the combined recruitment efforts, consent to participate could be successively achieved for 27 EMS staff members, three of which could not be reached later for reasons unknown. The interviewed sample consists of three female and 21 male paramedic EMS personnel. The majority of the sample had the higher-level paramedic certification of “Notfallsanitäter”. One participant, while also qualified as a paramedic, was currently assigned as an EMS dispatcher, while the remainder of the sample carried out regular clinical EMS duties. While some participants mentioned that their daily work did also encompass other Fire Brigade duties like firefighting, this was not systematically assessed in the documentation accompanying the interviews. Two participants reported working part-time. For more details on the sample’s characteristics, see Table 2.

**Table 2:** Characteristics of participating EMS personnel

		n (%)
**Sex**	Female	3 (12.5)
	Male	21 (87.5)
**Age groups** **(years)**	20–29	7 (29.2)
	30–39	9 (37.5)
	≥ 40	8 (33.3)
	Mean	36.6
	Median	34.5
	Range	22–55
**Professional experience in EMS** **(years)**	Mean	12.7
	Median	10
	Range	1–32
**Qualification level**	“Notfallsanitäter”	18 (75.0)
	“Rettungssanitäter”	6 (25.0)

Interviews had a mean duration of 32 minutes and were conducted in German. Presented citations from the interviews were translated to English from their original German language by the authors.

### Technical and organizational feasibility

The interviewed EMS staff voiced various conceivable facilitating factors concerning a direct patient redirection, but potential barriers were pointed out altogether more prominently. Overall, implementation was described as presumably very complicated.

#### Feasible, but only under certain conditions …

For some interviewees, a redirection of patients would be technically feasible within the existing infrastructure of the EMS. It was suggested that documentation for such outpatient cases could be performed on-site using EMS tablets, and partner practices could be integrated into the ambulance communication software (which is already used to communicate with hospitals and includes a digital encryption) without too much effort and resources. In this context, some participants stressed the need for the participating practices to provide regular updates of their current treatment capacities to avoid unnecessary waiting times and redundant transports.

*“We at the Berlin Fire Brigade have been documenting with tablets for a few years already [ …]* *Those deployment protocols [ …] can be sent with an end-to-end encryption via fax or e-mail [ …] to the primary care practice. On the technical side [ …] I do not see any problem here.”* (R1).

*“All these cooperating primary care practices could be integrated into the system, just like the hospitals.”* (R14).

*“There is this [ …] information system [ …] and we could check in advance whether it makes sense to drive there. [ …] The general practitioner would have to provide an hourly update about his remaining capacities.”* (R8).

It was repeatedly voiced in the interviews that certain organizational conditions would have to be met to ensure seamless feasibility of redirection. One participant recommended the development of a suitable SOP (Standard Operating Procedure) to assure a consistent process and documentation. Others estimated a need for increasing the quantity of ambulance vehicles and staffing paramedics. The necessity of a sufficient availability of large partner practices citywide was remarked in some interviews, which should ideally offer different specialities and out-of-hours care.

*“The organizational framework would have to be specified by the medical director and his SOP team [ …] and then this would be feasible [ …]*. *”* (R12).

*“Because [ …] we would need to acquire more ambulances, and these would have to be staffed appropriately.”* (R2).

*“[ …] if the [partner practices] would be in a central location, easily reachable for many vehicles, [ …] this would be well realizable.”* (R14).

*“There would have to be [ …] large practices [ …] capable to treat at 4 am in the night.”* (R10).

Some participants emphasized their professional competence to adequately assess patient care needs and decide on the appropriate care setting.

*“I, as a qualified paramedic, would be happy to take responsibility for this, and I feel quite capable to realize this.”* (R3).

According to several interviewed EMS staff, a preceding pilot test phase (e. g. in certain EMS stations) could help to evaluate feasibility.

*“A test phase would be required. It would be necessary to choose certain stations and certain vehicles [ …] for a defined period of time.”* (R22).

#### Actually not feasible...

Despite the depicted deliberations on necessary technical and infrastructural conditions voiced in some interviews, the majority of participants related the expectation that an implementation of the proposed direct patient redirection would be altogether impossible due to insurmountable organizational barriers. A central concern in this regard was the high current EMS workload associated with frequently stressed staff and lack of personnel and ambulance vehicles. It was presumed frequently that redirection would further increase workload (e. g. due to more time-consuming rides, especially in big cities, aggravated by a potential lack of authorization to activate vehicle blue lights and sirens due to the low-urgency patients transported), which could not be managed with the current resources. Some participants feared that this could result in EMS not being able to adequately take care of critically ill patients on time.

*“Currently this is [ …] not realizable with the personnel and vehicle resources available. This would currently be at the expense of regular prehospital emergency care [ …].*” (R5).

*“Then I drive through [ …] the big city without special permissions [blue lights and sirens] and I do have to wait out every traffic jam and am only driving around patients. I do not think this will work.”* (R4).

*“Ambulances then have longer distances to cover, and eventual real emergency patients will not get the help needed as fast.”* (R26).

Limited diagnostic options of the EMS were also addressed critically in the interviews. The assessment of patients’ care needs was described as a “snapshot” that could change within only a few hours, and interviewees voiced concerns for patient safety associated with bypassing regular emergency department care in this context.

*“You do not always know: what will the situation be in two or three hours? This can change at any instant [ …] Not every ambulatory case remains an ambulatory case.”* (R22).

Interviewees stressed the high existing caseload in ambulatory practice settings, and some assumed that patient redirection could lead to an overload of practices. The concern was voiced that this would further complicate practices’ appointment scheduling.

*“Will not work. [ …] Primary care doctors are partially overstrained already. [ …] Many physicians do not accept any new patients currently.”* (R1).

*“They schedule their patients [ …] and if these physicians would have to keep free appointment slots for eventual emergency patients, I do not know whether this is actually feasible.”* (R27).

In numerous interviews, the integration of other transport options was suggested as a potentially sensible option. It was proposed to assign certain patients to alternative non-EMS patient transfer services or the ambulatory on-call medical assistance service. The option of EMS redirecting potential outpatients to existing urgent care practices co-located with emergency departments was also mentioned.

*“Then we would have to get them [patient transport services and house call physicians] on board, integrate them into our dispatch system, and transfer this to them. They would then transport the patients [ …] to the practice. This would be a relief [ …] for EMS.”* (R8).

*“In the dispatch system, there should be the option to transfer to the statutory health insurance physicians’ urgent care service.”* (R5).

*“I [ …] thought it would be quite fine if patients could be just transported to the urgent care center on the hospital grounds.”* (R4).

### Acceptance by stakeholders

#### EMS personnel

Participants related thoughts regarding their own potential acceptance of a patient redirection scheme, and their conjectures regarding acceptance by EMS staff in general. However, these two aspects usually intertwined in the statements, and interviewees usually did not explicitly differentiate their own viewpoint from other colleagues’ potential acceptance. Statements in the interviews were quite heterogeneous, but most participants were rather opposed to the idea of redirection. This mirrors the multitude of barriers to implementation anticipated by the interviewees. However, positive aspects of redirection and facilitating factors for acceptance by EMS staff were also portrayed in the interviews. In that respect, the expected decreasing workload of EDs was positively highlighted by almost all interviewees, but many claimed that this would be the sole beneficial aspect. Some participants described the model as a good starting point which could serve to expedite further necessary changes in EMS practice and enhance paramedics’ scope for decision making. The expectation that younger EMS staff would be especially open to such changes was also voiced.

*“In general, this is a good idea that should be pursued. If this is an established procedure, it will serve to [ …] relieve emergency departments [ …].”* (R5).

*“We do not have this option to select at the moment, to [ …] bring a patient to his primary care doctor. Basically, to have the option [ …] would be a great asset.” (R2).*

*“There are some colleagues, the young [ …]*, *they would naturally be very keen for such things.”* (R12).

A few of the interviewed EMS staff members expected a potential reduction of conflicts between EMS and ED personnel as a result of reduced ED workload with accelerated patient flow and more time to focus on “real emergencies”.

*“A positive aspect is [ …] that emergency departments are able to take care of more real emergency patients and have enough time for these.”* (R18).

*“[ …] this would even improve the whole cooperation [with the emergency department] again.”* (R5).

Two interviewees regarded redirection as a potentially promising opportunity to initiate changes in the general public’s view. They surmised that if people experience that they are not automatically transported to the hospital by EMS with every conceivable medical complaint, they might initially consider ambulatory care as an alternative to EMS more frequently and decide to visit a practice instead of alerting EMS dispatch.

*“Maybe society will rethink and realize: I am not always brought to the hospital, but maybe to a practice instead. I could rather go to a doctor’s practice myself.”* (R14).

Despite a few proponents of the model stressing potential positive effects, most interviewed EMS staff assumed a low acceptance within the EMS, as already mentioned. In this context, interviewees were very frequently apprehensive of the expected increase in workload and associated negative effects on motivation and satisfaction of EMS personnel. Overall, while many indeed acknowledged a potential relief of ED burden, interview participants did not consider patient redirection to ambulatory care a viable solution to the problems of high EMS caseload and the constraints of scarce financial resources.

*“We are [ …] already at the limit. Beyond the limit. I do not think it will further employee motivation of EMS colleagues.”* (R19).

*“Naturally, the Fire Department’s problem will not be solved, as people will continue to call.” (R3).*

*“[ …] one of our core problems, the immense costs. This will not be solved, because an EMS deployment will still happen.”* (R13).

Transportation of patients without urgent acute care needs to any care facility was described as intrinsically unnecessary by almost all interviewees. From their point of view, such patients could rather organize their way to an ambulatory care consultation on their own, e. g. by public transportation. The sentiment that transportation of patients with non-hospital care needs actually leads the meaning and purpose of EMS work astray and is therefore disruptive for paramedic personnels’ motivation was quite prevalent. Participants repeatedly expressed their profession’s mission as taking care of “real emergencies” rather than being misused as kinds of taxi or patient transport drivers.

*“I believe most paramedics do what they consider meaningful. As I said, covering this whole primary care area, this does not make sense to anyone.”* (R10).

*“I understand EMS as [ …] patients who are in dire need of urgent help [ …]. [ …] I believe acceptance will fail because people will say, “Why does somebody drive to a general practitioner with EMS, as he could alternatively take the taxi or bus or just walk?” We could then just paint the word taxi on our vehicles.”* (R19).

In addition, some participants feared that redirection to practices may actually introduce an improper appeal of consulting EMS for mere needs of transportation to ambulatory care providers.

*“You just have to pay attention not to add an incentive for people using this as a taxi to the general practitioner or other practices or as an invitation to do so.”* (R3).

#### Patients

While they were tendentially rather reserved towards redirection schemes themselves, interviewed EMS staff frequently expected that patients would be conceivably open minded regarding the proposed concept. It was surmised that patients would appreciate conveniently receiving help in the suitable setting, potentially associated with shorter waiting times. The importance of ensuring transparency and creating trust by comprehensive information was frequently stressed in the interviews.

*“I believe patients would like this, to be brought to a practice, because they do not have to wait there for so long.”* (R4).

*“I think if proper information is provided [ …], acceptance will be quite high on the patient side.”* (R18).

However, a few participants also expressed contrasting opinions of expected low acceptance by patients, with high expectations of EMS callers regarding possible diagnostics mentioned as an important reason.

*“I believe patients want to go to a provider and get an all-inclusive package. Meaning they want a lab test [ …] and an x-ray, on the same day.”* (R1).

Table 3 gives a condensed overview of the key results of the interview study.

**Table 3:** Themes, interview guide questions and paramedic quotes

Subject	Interview guide question	Themes addressed in interviews	Representative paramedic quote (abridged)
**Technical and organizational feasibility**	What do you think about the redirection model proposed by the Government Advisory Council? What aspects of the model are especially positive or negative in your view? How do you assess the technical and organizational feasibility of this pathway? Could you give reasons for this judgment?	**Feasible, but only under certain conditions …**	
		Documentation / communication	*“On the technical side […] I do not see any problem here.”*
		Process	*“[…] we would need to acquire more ambulances, and these would have to be staffed appropriately.”*
		Paramedics’ competence	*“I, as a qualified paramedic, would be happy to take responsibility […]*”
		**Actually not feasible …**	
		EMS workload	*“[…] not realizable with the personnel and vehicle resources available.”*
		Patient safety	*“Not every ambulatory case remains an ambulatory case.”*
		Practice workload	*“Primary care doctors are partially overstrained already.”*
		Alternative options	*“[…] there should be the option to transfer to the statutory health insurance physicians’ urgent care service.” (R5)*
**Acceptance by stakeholders**	How do you appraise the acceptability by EMS staff/patients? Could you give reasons for this judgment?	**EMS personnel**	
		EDs relieved	*“[…] emergency departments are able to take care of more real emergency patients […]”*
		No benefit to EMS	*“[…] people will continue to call.”*
		Potential misuse	*“[…] incentive for people using this as a taxi to the general practitioner […]”*
		**Patients**	
		Attractive	*“[…] patients would like this, […] because they do not have to wait there for so long.”*
		Unattractive	*“I believe patients want to go to a provider and get an all-inclusive package.”*

## Discussion

### Summary of findings

Statements appraising feasibility of a direct patient redirection from EMS to ambulatory care practices positively were mainly about technical realizability, e. g. linkage of software platforms, and a few interviewees expected only little effort required to implement a working infrastructure. Potential prerequisite conditions mentioned included an extension of EMS capacities or a comprehensive accessibility of suitable practices. However, the majority of interviewees rather assessed patient redirection as organizationally impossible. It was prevalently expected that the potentially increasing workload could not be managed with the present resources regarding staff and ambulance vehicles. Participants even feared that “real emergencies” may be put at risk if EMS personnel would be tied up in transporting cases not really needing emergency care. Interviewees did prevailingly advocate the integration of other transport options for less urgent cases. Another patient safety concern voiced was that some cases with unrecognized serious conditions may be wrongly relocated to practices, due to limited diagnostic options in the EMS environment.

Many participants welcomed the notion to relieve EDs and their staff and to free ED resources for “real emergencies”. However, they conversely expected undesirable effects for EMS and thus voiced potential low acceptance, both from a personal viewpoint and with regard to paramedic personnel in general. Negative consequences feared were mainly increased workload and associated dissatisfaction among the staff. The sentiment of EMS potentially being used as a taxi service by having to provide “unnecessary” transport to outpatients was prominently raised. Concerning the presumed perspective of patients, interviewees expected a high level of acceptance if informed properly. Advantages like available transport and shorter waiting times would also serve to boost patients’ openness to redirection, while also entailing a danger of misuse for pure convenience reasons.

### Results in context

For the interviewed EMS staff, the attractiveness of patient redirection concepts is mainly rooted in a potential relief of ED workload by shifting patients to ambulatory structures. From the hospital perspective, this is frequently considered an important and necessary step towards ensuring appropriate care for “real emergencies” in the ED setting [22]. Some have argued in this context that this patient group uses only small proportions of ED resources [23] and that their impact on waiting times is limited [24]. However, this is discussed controversially and may depend on specifics of the setting studied. As less urgent patients constitute a large proportion of ED consulters [25, 26], it appears very plausible that redirection schemes would serve to diminish pressure.

It was suggested in the interviews that use of non-EMS ambulance transports for redirection would be a far better option for less urgent cases, as resources of both EDs and EMS would be freed. However, while this would ostensibly relieve workload of emergency care providers, the safety of such notions depends on the quality and reliability of the alternative service in question. Non-emergency patient transports have been described as potentially prone to insufficient standardisation and poor communication processes [27]. The topic of “patient safety” also became apparent in the context of time resources and diagnostic options of the EMS. Interviewed staff expressed concerns about the care for time-critical patients, which could be endangered due to resources tied up by redirection, and also about potential misclassification of cases as non-urgent. As urgency categorization has been described as potentially unreliable even in the context of usual hospital-based triage [28], it appears reasonable that this would also – or even more – apply to prehospital care. Research in this context however has shown heterogeneous results. Several studies indicated that the urgency of prehospital patients may be underestimated [12, 29], and that ambulance staff lack confidence in determining patients’ care needs [30, 31]. In contrast, in a UK study from 2008 [32], EMS staff were able to estimate the likelihood of patients’ admission with an adequate accuracy. Some participants of the interviews also talked positively about their ability for decision-making. One participant expressed the view that implementation would be possible with the development of an appropriate SOP. Results of a prospective study by Pointer et al. [33] however showed that EMS staff using written guidelines do not achieve an acceptable level of triage accuracy to determine disposition of patients in the field. This question thus remains altogether unresolved scientifically, and as paramedics’ qualifications and professional training vary internationally, the setting may play an important role here, too.

In the interviews, lack of resources in the EMS and outpatient sector was frequently criticised, and arguments related to resource scarcity prominently feature in the statements on both doubtable feasibility and low acceptance of redirection. Even participants principally acceding to a potential feasibility of the scheme stressed the importance of a prerequisite expansion of personnel and structural capacities (such as nationwide availability of large medical practices). Considering the literature, interviewees’ appraisal of resource availability as a core problem area seems accurate: the logistical effort involved in implementing patients’ redirection and the potentially high investment costs, e. g. for additional staff or training, have been likewise described by others [3, 12]. An additional factor potentially boosting expenditure was also raised in the interviews: prevalent use of EMS as a convenient taxi service (termed “a wrong incentive”) would certainly constitute an economically wasteful situation.

In context of the acceptance of patient redirection, interviewed EMS staff tried to put themselves into different stakeholders’ position and related their views regarding respective benefits and drawbacks, based on their professional and personal experiences with a high number of patients. In the interviews, both individual acceptance of the interviewee and presumed acceptance by other EMS colleagues were described or assessed as rather low. In this context, the unwanted situation of an “unnecessary” transport of outpatients was mentioned very prominently. Participants clearly felt that being used as a mere ambulance transport or even a taxi would equal a vilification of their job and qualification, and the notion that non-emergency patients should go and seek appropriate outpatient care on their own was raised. However, a survey of walk-in ED patients showed that patients sometimes do not know alternative care options for acute problems [34]. In line with this, a qualitative investigation of ED outpatients conducted in our network suggested limited health competencies regarding self-assessment of complaints and the associated urgency, with resulting decision-making difficulties in acute situations [35]. It is difficult to judge whether the prominently raised apprehensions regarding potential misuse of EMS constitutes an adequate assessment or rather an indication of a prevalent general resistance to change and expansion of the profession’s scope, and views speculatively may change in the wake of establishing new care pathways and paramedics’ functions [36]. A stronger interlocking of primary care and EMS care may both shift part of the care burden from hospital structures to the ambulatory sector and altogether increase low-acuity utilization by supply-induced demand if the new care pathways are attractive to patients [37].

In this context, most of the interviewed personnel supposed that patients would accept the redirection to other care facilities if provided an appropriate elucidation. Validity of this expectation is difficult to judge, as the pertinent literature is comparably scarce and controversial. In line with our participants’ valuation, a study by Jones et al. [38] suggested that patients find alternative destinations and transport for acute care acceptable. Willingness to consider such alternatives however is difficult to predict and potentially depends on the perceived individual benefits. Contrarily to the results of this study and the expectations in our interviews however, others have reported negative attitudes of ED patients towards possible redirections [39, 40]. Interestingly, interviewees in our study clearly described potential benefits to patients (e.g. shorter waiting times) as well as EDs, but nevertheless were prevailingly aversive to redirection concepts as to the potential negative consequences to their own workload, work environment, and professional mission. Such priority setting however should not be interpreted as self-centeredness, but as an indication of a profession frequently feeling strained [41–43] – as formidably illustrated by the statement “we are [ …] already at the limit. Beyond the limit.” (R19). Correspondingly, paramedics have scored significantly lower than the mixed-profession reference group in a recent nationwide job satisfaction survey [44]. It thus appears reasonable that readiness for change is limited as to apprehension towards potential new burdens.

### Strengths and limitations

To our knowledge, this study constitutes the first qualitative investigation of paramedics’ perspectives on potential EMS patient redirection to ambulatory care. The qualitative interviews with EMS personnel add a new perspective to the discussion about alternative pathways for patients with non-hospital care needs and allow a more comprehensive assessment of the redirection potential. Studies purely looking at EMS patients’ medical case characteristics and labelling medically less urgent cases as “actually eligible for primary care” fall short of considering the implications of changing care pathways, of associated facilitators and barriers, and of potential consequences for the stakeholders involved.

As to limitations, we must stress that a convenience sampling was chosen due to the initially expected limited interest in participation, which could have biased the spectrum of views captured due to self-selection. The sample is balanced in regard to age groups; however, men are clearly over-represented, reflecting the gender imbalance in EMS staff. Nevertheless, we did not get the impression of female participants’ views tendentially differing from the male majority when performing our analysis.

Participant statements on the acceptance by EMS staff, and even more so by patients, may be associated with assumptions and presumptions of the interviewees and therefore must be regarded with a considerable degree of caution, as they constitute a second-hand view. Nevertheless, valuations of potential acceptance voiced by the EMS staff members are valuable, as these are not ungrounded utterances, but insights rooted in many years of professional experience and permanent contact with the colleagues, as well as numerous patients.

As to the study objective focused on the current political discussion, we did only present the redirection model proposed by the Advisory Council to the participants for consideration, and – while being more speculative – an inclusion of variants or alternative models in the interview guide could potentially have generated further insights and increased generalizability.

Lastly, the interviews contain specific details of the German health care system that may not be transferable to other health care systems.

## Conclusions

This study investigated EMS staffs’ assessment of feasibility and acceptance of a notion of directly redirecting lower-urgency EMS to ambulatory care in the context of the German health care system. Despite potential benefits of redirection for EDs and patients, interviewees depicted numerous barriers regarding both organizational feasibility and acceptance by EMS personnel. Besides logistic challenges and necessary expenditure, participants’ tendentially negative assessment seems to arise from already strained EMS resources and associated reluctance to change, augmented by concerns of patient safety. It thus seems very important that potential health care reforms including prehospital patient redirection consider paramedics’ perspective and provide measures and resources to ascertain both the operative readiness of EMS and the job satisfaction of its personnel, as they are vital stakeholders for any new pathway’s success.

## Supplementary Information

**Additional file 1.** Interview guide for paramedic interviews in the EMAPREPARE study

## Data Availability

Data generated and analyzed during the study (audio files and transcripts) is not publicly available for reasons of confidentiality. Individual reasonable requests for data should be addressed to the corresponding author.

## Abbreviations

ED: Emergency department
EMS: Emergency medical services
PC: Primary care
EMANet: Emergency and Acute Medicine Network for Health Care Research Berlin
EMAPREPARE: Emergency and Acute Medicine - Primary Care Demands in Patients resorting to Emergency Departments
SOP: Standard Operating Procedure
UK: United Kingdom
