# Supplementary material for: Redirecting emergency medical services patients with unmet primary care needs: the perspective of paramedics on feasibility and acceptance of an alternative care path in a qualitative investigation from Berlin, Germany
Source: BMC Emerg Med. 2022 Jun 11;22:103. doi: 10.1186/s12873-022-00660-2 (PMC9187922; doi:10.1186/s12873-022-00660-2)
Supplement: Supplementary file 1 — Additional file 1. Interview guide for paramedic interviews in the EMAPREPARE study [file 12873_2022_660_MOESM1_ESM.pdf]

Additional file 1: Interview guide for paramedic interviews in the EMAPREPARE study

Thank you very much for taking the time for this interview. My name is… and I am a researcher at the Institute of General Practice at Charité University Medical Center.

I will ask you some questions concerning your professional experience with patients with an acute, but non-urgent care need. Additionally, I am interested in your views about a care model proposed by the Government Advisory Council that involves redirection of such patients to a primary care setting. You did already receive the study information, but I will be glad to provide further information if required. Please relate your views and everything that comes to your mind freely and openly, I will ask more questions if necessary. Some questions may seem repetitive or similar to each other, please do not be surprised. You can answer all questions in your own time, there are no right or wrong answers. I am interested in your individual perspective. Your views and experiences are important to introduce the health care provider perspective into the current political discussion.

Do you have any remaining questions concerning the study information or data protection? Otherwise, I will now start recording the audio.

**Lead question**	**Follow-up question**	**Control question**
Please tell me about an EMS case you remember, in which the patient in your opinion had an ambulatory care need		How exactly did this happen?<br><br>Could you provide some more detail?
In your view, why do patients with ambulatory care needs call EMS?	Which motives do you encounter most frequently in your daily work?	Could you elaborate on this?
Which groups of patients do call EMS for ambulatory care needs especially frequently?	Why do these groups particularly call EMS?	
In your view, how has EMS utilization for ambulatory care needs evolved in the past five years?	Did your colleagues voice an opinion in this context?	
Which consequences do you notice in your daily work if patients call EMS with ambulatory care needs?	How do you and your colleagues cope with this?	Could you tell us more about your reasons for this view?
What measures would you consider appropriate to manage this situation?	Could you imagine any further measures or changes in this context?	
By the following questions, I would like to learn more about your view on the proposed redirection of patients with acute, but non-urgent care needs into an ambulatory care	If necessary, explain the model proposed by the advisory council:<br><br>Redirection of patients appearing to have an	

setting, particularly primary care. Do you need additional information about this scheme, or do you feel sufficiently informed about this?	ambulatory care need when evaluated on-site into ambulatory / primary care practices by EMS transport<br><br>Possibility to check treatment capacities in cooperating practices online real-time, allowing for direct redirection<br><br>Model aims for a relief of ED burden, combined with targeted patient care and resource use	
What did first come to your mind when you learned about this notion?		
What do you think about the redirection model proposed by the Government Advisory Council?		
From your professional experience, how do you assess the technical and organizational feasibility of this pathway?	What aspects of the model are especially positive or negative in your view?<br><br>How do you asses organizational feasibility in the context of EMS operations?	Could you give reasons for this judgment?
How do you appraise the acceptability by EMS staff / patients? / medical practices?		Could you give reasons for this judgment?
Should anything be done to prevent these patients from calling EMS?	Why?	Could you elaborate on this?
Now we are nearing the end of the interview. Is there anything further you would like to add concerning the topics mentioned, or is there anything you would consider important that has not been addressed?<br><br>I would now like to inquire about some demographic and work-related details that are important for our analysis.<br><br>Thank you very much for your participation!		
